# Effect of High Ammonium Salt Concentration and Temperature on the Structure, Morphology, and Ionic Conductivity of Proton-Conductor Solid Polymer Electrolytes Based PVA

**DOI:** 10.3390/membranes10100262

**Published:** 2020-09-28

**Authors:** Maryam A. M. Saeed, Omed Gh. Abdullah

**Affiliations:** 1 Department of Physics, College of Science, University of Mosul, Mosul 41002, Iraq; alhabishmeryem@gmail.com; 2 Advanced Materials Research Lab., Department of Physics, College of Science, University of Sulaimani, Sulaymaniyah, Kurdistan Region 46001, Iraq

**Keywords:** proton conductor, polymer electrolyte, ionic conductivity, arrhenius, VTF, CBH

## Abstract

Polyvinyl alcohol (PVA) based proton-conducting solid polymer electrolyte was prepared with a high salt concentration of ammonium nitrate (NH_4_NO_3_) by the technique of solvent casting. From the X-ray diffraction studies, the semicrystalline nature of PVA with the inclusion of NH_4_NO_3_ was studied. XRD analysis indicates that the highest ion conductive sample exhibits the minimum crystalline nature. The decreasing trend of Jonscher-exponent with temperature rise reveals that the present system is insured by the correlated barrier hopping (CBH) model. The maximum room temperature conductivity was found to be 5.17 × 10^−5^ S/cm for PVA loaded 30 wt.% of NH_4_NO_3_. The ionic transport of the proton-conducting solid polymer electrolyte was studied at the temperature range of 303–353 K. The conductivity-temperature relationship of the systems was analyzed using both the Arrhenius and Vogel–Tammann–Fulcher (VTF) models to explain the ionic hopping mechanism for the system.

## 1. Introduction

Solid polymer electrolytes (SPEs) are crucial compounds having promising applications for electrochemical devices, such as high powered solid-state rechargeable batteries, supercapacitors, chemical sensors, electrochromic displays, and fuel cells due to several advantages over the gel and liquid electrolytes [1,2,3,4]. The principal advantages of SPEs are their good mechanical properties, adaptability, wide electrochemical accuracy, simplicity of manufacture thin films, and flexibility [5,6]. The SPEs are characterized as ionic conducting polymer materials that are sophisticated by dissolving appropriate salt in the high molecular weight polar polymers.

Over the past decades, SPEs incorporated with lithium salts are prepared excessively due to an abundance of lithium salts, small Li-ion radii, and being lightweight. They are systematically investigated to reveal the possibility of making lithium-ion rechargeable batteries with high specific capacity and high electrochemical reduction potential [7]. Nowadays, proton-conducting SPEs have gained more attention due to their extensive applications in electrochemical sensors, reactors, and proton-exchange membrane fuel cells [8,9]. A literature survey reveals that incorporating ammonium salts into polar polymer produces proton-conducting electrolyte, due to one loosely bound of four protons attached to nitrogen [8,10].

Many researchers have already studied proton-conducting SPEs by incorporating ammonium salts to different polar polymers such as polyvinyl pyrrolidone, polyethylene oxide, polyvinyl alcohol, polyvinyl chloride, and polyacrylonitrile [11,12,13]. From the literature study, polyvinyl alcohol (PVA) has attracted great attention due to its good film-forming, good mechanical strength, biocompatibility, biodegradability, and nontoxicity [14,15]. PVA is a hydrophilic, synthetic, dielectric, semicrystalline polymer material at room temperature, which is widely used as a host matrix in the various SPE systems due to its durable polar nature [5,16]. The electrical conductivity of PVA was enhanced by some researchers upon the addition of different ammonium salts to prepare proton (H^+^)-conducting SPEs. Radha et al. [17] reported the highest ionic conductivity at ambient temperature in the order of 10^−6^ S/cm for PVA/NH_4_F proton-conducting SPEs. Hema et al. [18] reported the enhancement in ionic conductivity value in the order of 10^−3^ S/cm for PVA/NH_4_I, 10^−4^ S/cm for PVA/NH_4_Br, and 10^−5^ S/cm for PVA/NH_4_Cl proton-conducting SPEs. In this work, series of proton-conducting SPEs are prepared from PVA as the host matrix, and the low-cost ammonium nitrate (NH_4_NO_3_) is used as a proton source for improving the ionic conductivity of the system. The structural, electrical, and transport properties of the prepared samples are studied as a function of salt concentrations, frequency, and temperature to expect the possible applications of the system.

## 2. Materials and Methods

Polyvinyl alcohol (PVA), low molecular weight, 98–99% hydrolyzed, purchased from Alfa Aesar (Kandel, Germany), and ammonium nitrate (NH_4_NO_3_), Mw = 80.04 g/mol, purchased from Merck (Darmstadt, Germany), were used as raw materials. Double-distilled water was used as a solvent in the experiment.

To set up a series of high salt concentration proton-conducting SPE films loaded with 10, 20, 30, 40, and 50 wt.% of NH_4_NO_3_, 2 g of PVA was dissolved in 40 mL double-distilled water separately, and the appropriate amount of salts dissolved in 5 mL were mixed together under stirring at room temperature until viscous homogeneous mixtures were achieved. The final polymer solution was cast in PP Petri dishes and dried slowly at the ambient temperature for the films to form. Since PVA has a hydrophilic nature, to ensure dryness of the films, the samples were kept in a dust-free chamber with silica gel for 14 days before being used in experiments. The flat, coherent films had been achieved and set aside in desiccators prior to characterization. The proton-conducting SPE films were coded as SPE-10, SPE-20, SPE-30, SPE-40, and SPE-50, for PVA incorporated, respectively, with 10, 20, 30, 40, and 50 wt.% of NH_4_NO_3_ salt.

Structural behavior of the prepared proton-conducting SPEs are characterized by X-ray diffractometer made by Bruker D8 (Karlsruhe, Germany) with Cu-Kα radiation (λ = 1.5418 Å) with an increment of 0.02° in the range of 10°–70°. The change in surface morphologies of the prepared films was examined using JEOL JSM-6060 scanning electron microscopy (SEM (Tokyo, Japan)) at an accelerating voltage of 20 kV. Prior to imaging, all samples were coated with a thin gold layer by a sputtering process. Impedance spectroscopy analysis was carried out using KEYSIGHT E4980A LCR Meter (Santa Rosa, CA, USA) within a frequency range of 100 Hz–2 MHz in the temperature range of 303–353 K.

## 3. Results and Discussion

### 3.1. X-ray Diffraction Studies

**Figure 1:**
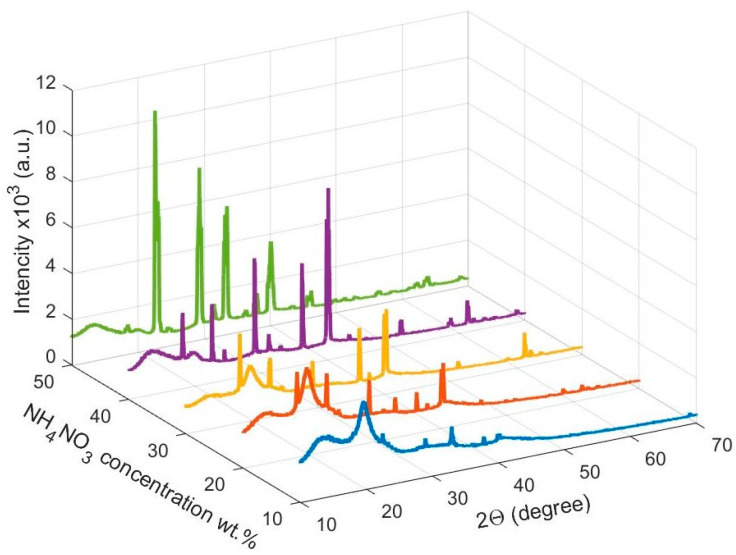
XRD pattern plots for PVA/NH_4_NO_3_ proton-conducting SPE films.

The structural properties of pure PVA and PVA/NH_4_NO_3_ proton-conducting SPE films were evaluated utilizing X-ray diffraction (XRD) studies and depicted in Figure 1. In the case of pure PVA, a broad, intense peak observed at a scattering angle centered at 2θ = 19.25°, indicate the semicrystalline structure of PVA [19,20]. The occurrence of the semicrystalline structure of PVA mainly arose due to a strong intramolecular and intermolecular hydrogen bonding between PVA molecules [21]. The intensity of the characterized peak of PVA was gradually decreased with increasing salt content and disappeared at high salt concentrations, which implies the changes that occurred in the semicrystalline phase of the PVA matrix, due to the interaction between hydroxyl groups of the PVA and cations of the dissolved salt [22,23].

**Figure 2:**
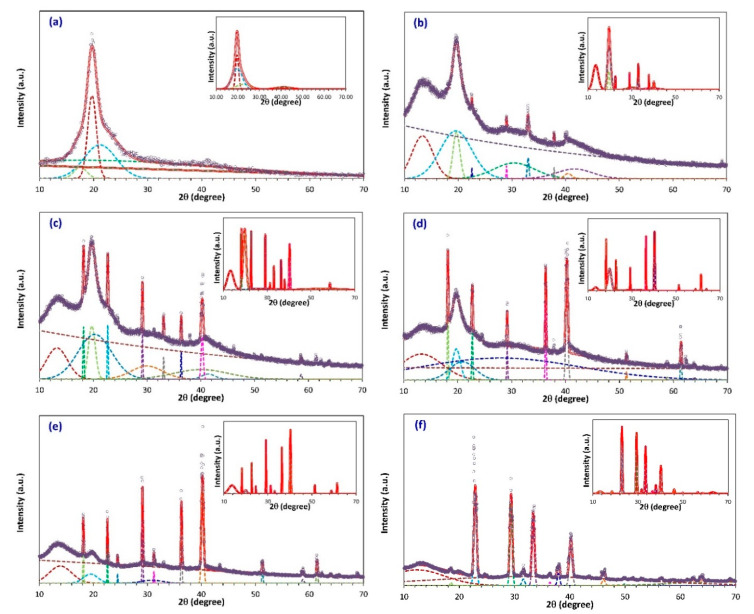
Represented the deconvoluted XRD peaks for (**a**) pure PVA, (**b**) SPE-10, (**c**) SPE-20, (**d**) SPE-30, (**e**) SPE-40, (**f**) SPE-50. The graphs show the peaks for both the crystalline and amorphous regions. The inner graph depicts the crystalline peaks only. (The solid lines represent the sum of the Gaussian fits in dotted lines).

The PVA/NH_4_NO_3_ proton-conducting SPE films show some sharp diffraction peaks, which depict the crystalline structure of NH_3_NO_3_ according to the Joint Committee on Powder Diffraction Standards (JCPDS) card No. 83-0520. [24]. This indicates the presence of some undissociated NH_4_NO_3_ salt due to the high salt concentration in the present proton-conducting SPE samples. The increase in the relative intensity of the characteristic peaks of NH_3_NO_3_ in the SPEs with increasing salt concentration was attributed to ion association and salt aggregation at the surface of the film, as described by Shuhaimi et al. [25]. A relationship between the intensity of the XRD peaks and the crystallinity has been reported by numerous researchers [26,27]. The percentage of crystallinity ($\chi_{c}$) was calculated from the following relationship [28,29];

(1) $$\chi_{c}=\frac{A_{c}}{A_{t}}\times 100\%$$

where $A_{c}$ and $A_{t}$ are the area under the crystalline peaks and the total area of the diffractogram, respectively, which were determined from the deconvolution of the XRD spectra using Fityk software [30]. The XRD pattern was fitted by Gaussian function mode. The deconvolution of the XRD peaks for pure PVA and PVA/NH_4_NO_3_ proton-conducting SPE films shown in Figure 2 and the calculated value of $\chi_{c}$ is displayed in Table 1. The outer graphs in Figure 2 show the deconvoluted peaks for both the crystalline and amorphous regions, which was used to determine $A_{t}$, whereas, the inner graph depicts only the crystalline peaks, which was used to calculate $A_{c}$. It can also be observed that the hump of pure PVA spectrum is deconvoluted into four peaks, whereas the XRD spectra of SPEs were deconvoluted into 10–14 peaks, depending on the number of crystalline peaks appearing in the XRD pattern, in order to obtain a more accurate estimate of the crystallinity percentage of the system.

It can be inferred from Table 1 that the sample containing 30 wt.% NH_4_NO_3_ exhibits the minimum degree of crystallinity (highest amorphous nature). It has been well reported that the ionic conductivity of SPEs increases with the increase in the amorphous domains [31,32]. Thus, it can be predicted that the proton-conducting SPE sample containing 30 wt.% NH_4_NO_3_ exhibits the highest electrical conductivity at room temperature.

### 3.2. Morphological Study

**Figure 3:**
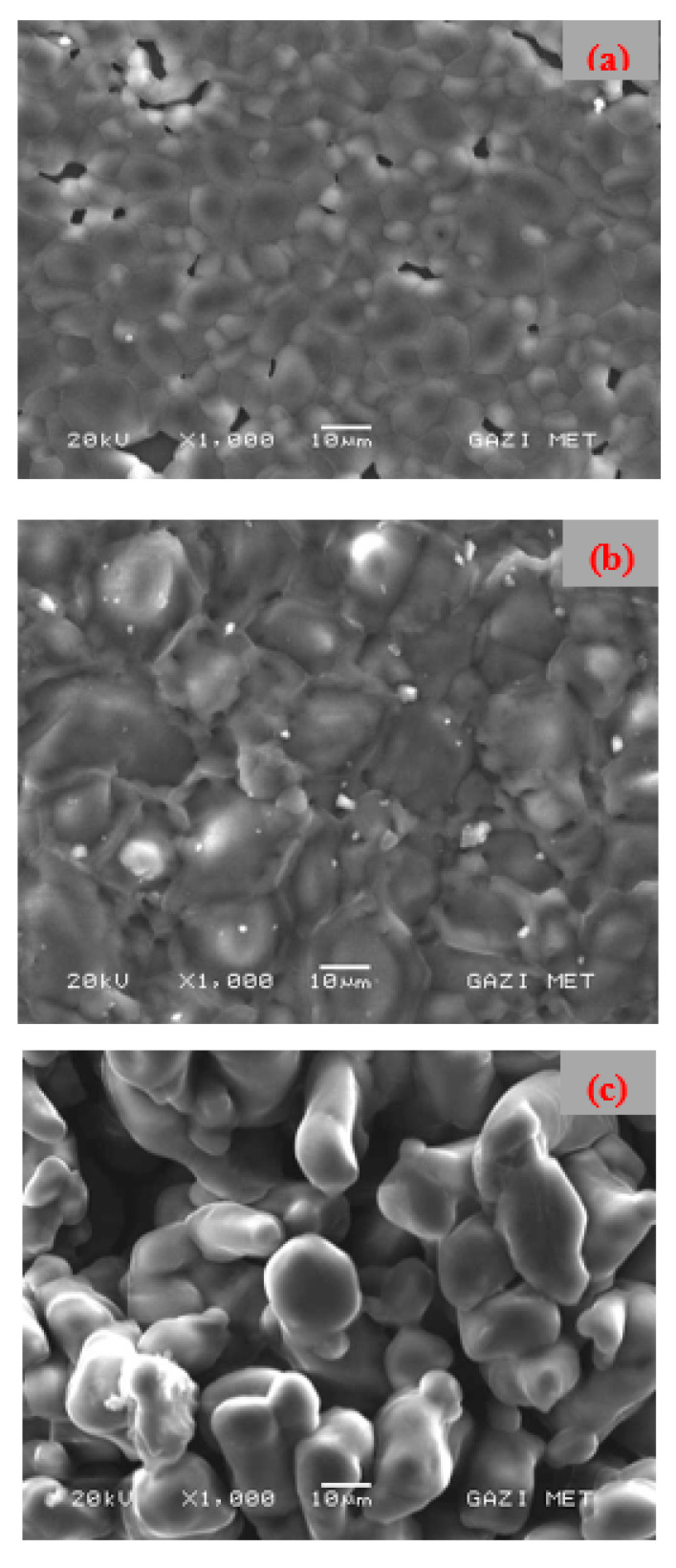
SEM micrographs of SPEs with different salt concentrations: (**a**) SPE-10, (**b**) SPE-30, and (**c**) SPE-50.

The surface morphology images for the prepared PVA/NH_4_NO_3_ SPE films with different salt contents are shown in Figure 3. The sample with low salt concentrations shows almost smooth and homogeneous surface morphology, while the much rougher surface texture was perceived for the high salt concentration sample (SPE-50). It is well reported in the literature that the enhanced surface roughness of the SPEs at high salt concentration mainly occurs due to the salt aggregation out of the surface [33,34]. Thus, at high salt content, the dissociated ion pairs begin to recombine to form a neutral salt and then salt aggregations out of the surface of the SPEs. Kadir et al. [35] also reported a similar observation for chitosan-polyethylene oxide doped NH_4_NO_3_.

According to the previous reports [36,37], the conducting ions move more freely in the electrolyte with smoother surface morphology and therefore caused an overall enhancement in conductivity of SPEs. Some researchers successfully established a correlation between surface roughness and DC conductivity [33,36]. They conclude that the crystalline aggregations of undissolved salt out of the film surface at high salt content caused a significant decrease in the number of charge carriers, and hence lead to the reduction in DC conductivity of the system [38].

### 3.3. Electrical Conductivity Studies

#### 3.3.1. Cole-Cole Plot

**Figure 4:**
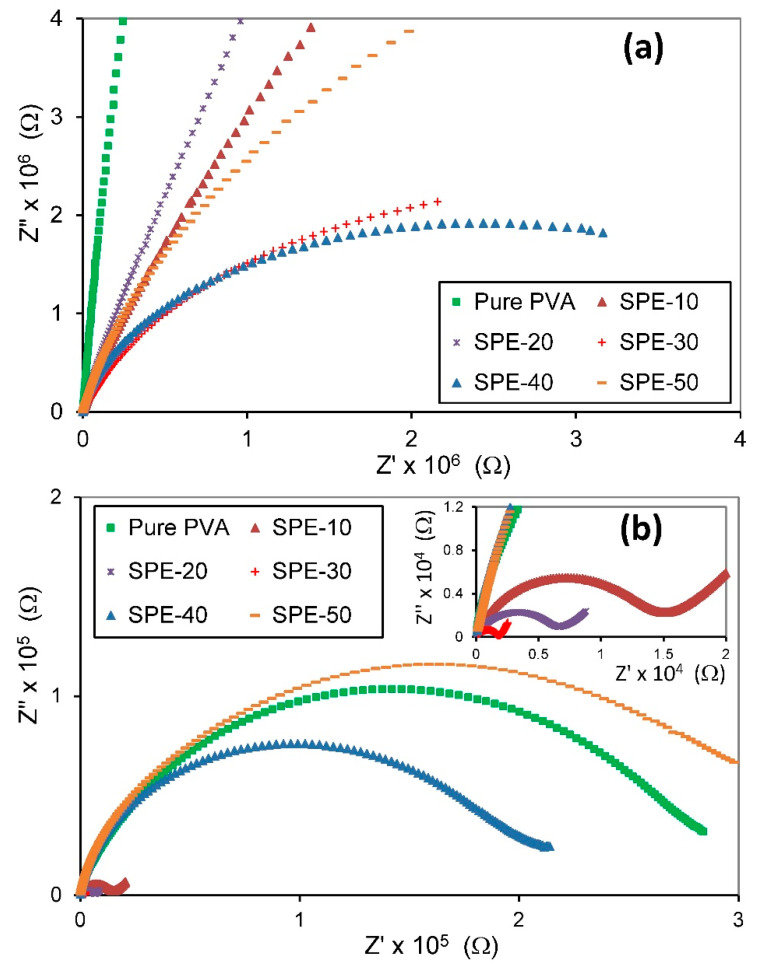
Cole-Cole plots for pure PVA and PVA/NH_4_NO_3_ proton-conducting SPE films at temperatures (**a**) 303 and (**b**) 353 K.

Figure 4 shows the relation of the real part (Z’) and the imaginary part (Z’’) of complex impedance for pure PVA and PVA/NH_4_NO_3_ proton-conducting SPE films with different concentration of NH_4_NO_3_ at two temperatures, 303 and 353 K. The Cole-Cole plots for PVA/NH_4_NO_3_ proton-conducting SPE films showed one semicircle arc that is followed by a spike at high temperature, which indicates a parallel combination of resistance and a capacitance in series [39]. Monitoring of a single semicircular arc for all SPEs is an indication of only one type of relaxation strategy existing in this system [40]. The intercept of the semicircle arc in the Cole-Cole plot with the real axis at the lower frequency end is usually used to determine the bulk resistance of SPE.

It is evident from Figure 4 that the change in salt concentrations and temperature plays a role in affecting the complex impedance spectra. At room temperature (Figure 4a), the complex impedance spectra have the same form for all SPE samples. However, at high temperature (Figure 4b), across the high concentration of NH_4_NO_3_, both high-frequency semicircular arc and low-frequency tail is observed. It is also evident that all the curves exhibit a tendency to bend towards the abscissa, and the radius of the semicircular arcs decreases with increasing temperature, denoting the increase in conductivity value with increasing temperature. Similar trends have been observed by many researchers for various SPE systems [41,42].

#### 3.3.2. Frequency-Dependent Conductivity

**Figure 5:**
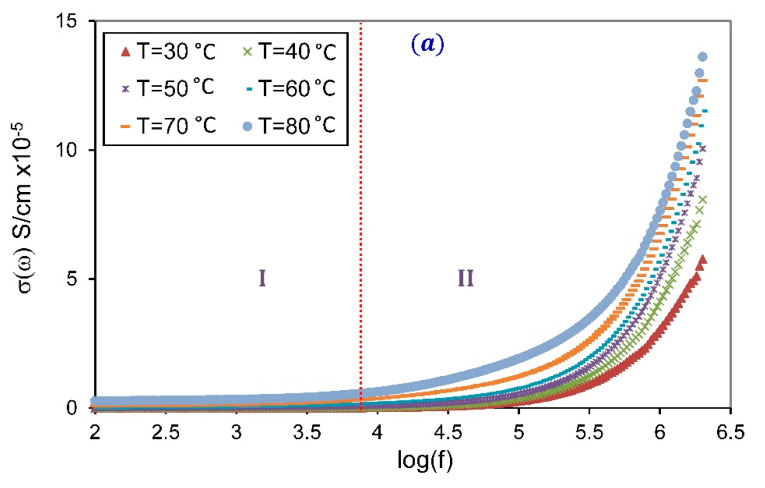

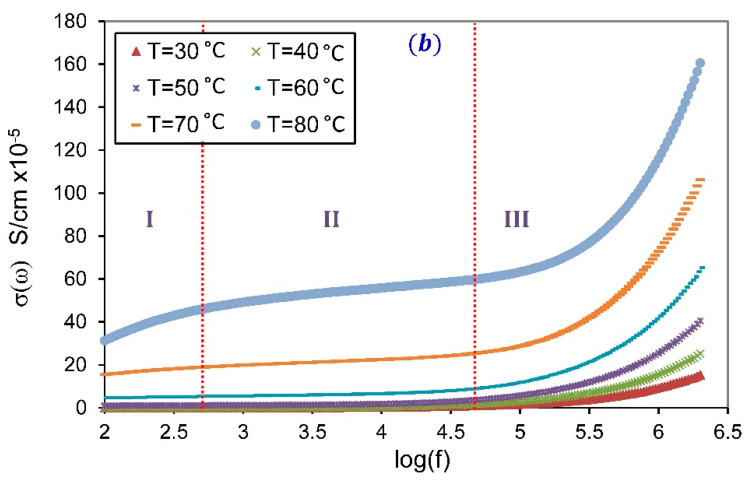
Conductivity versus log(f) for (**a**) pure PVA, (**b**) SPE-30, at different temperatures.

The study of electrical conductivity spectra for SPE systems at various temperatures provides us with significant information about the mechanisms of ionic conduction in the SPE materials [43]. The measured complex impedance of electrolyte was used to compute electrical conductivity (σ(ω)), using [44]:

(2) $$\sigma \left(\omega \right)=\frac{d}{A}\frac{Z^{\prime }}{\left(Z^{\prime 2}+Z^{\prime \prime 2}\right)}\;$$

where d and A are the thickness and the cross-section area of the sample, ω=2πf is the angular frequency. The spectra of σ(ω) for pure PVA and SPE-30 at various temperatures are presented in Figure 5. It is clearly seen that the spectra of pure PVA can be divided into a frequency-independent plateau region at low frequency, and a frequency-dependent dispersion region at high frequency. However, the conductivity spectra for SPE-30 can be divided into three regions; a low-frequency dispersion region, a frequency-independent plateau region in the mid-frequency region, and a high frequencies dispersion region. The low-frequency dispersion region is attributed to the space-charge accumulation at the electrode-specimen contact. The frequency-independent plateau region ascribed to the DC conductivity ($\sigma_{DC}$). The high-frequency conductivity dispersion region is expressed by Jonscher’s empirical power law:

(3) $$\sigma \left(\omega \right)=\sigma_{DC}+B\omega^{S}$$

where B is a pre-exponential factor, and S is the dimensionless power-law exponent, generally varies in the range 0<S<1 [45]. It is well established that the relation between S and temperature can be used to define the type of ion conduction processes that occur in the SPE due to the hopping of charge carriers over potential barriers in the system [14].

**Figure 6:**
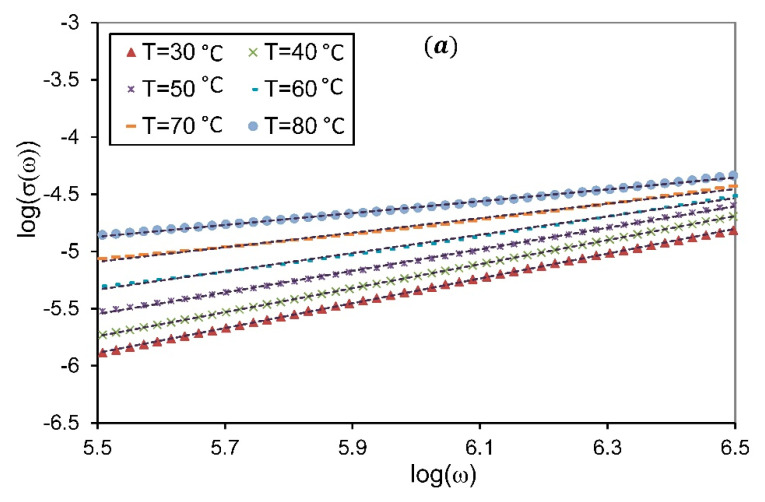

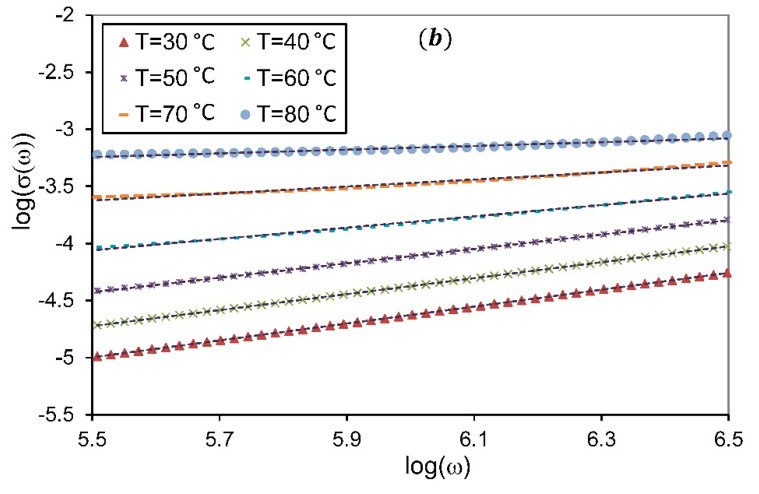
Plot of logσ(ω) versus logω for (**a**) pure PVA, (**b**) SPE-30, at different temperatures.

**Figure 7:**
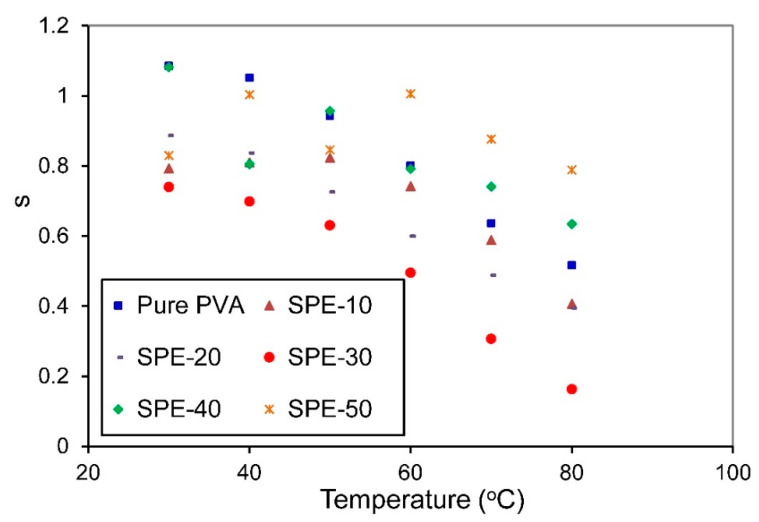
Variation of exponent S with temperature for PVA/NH_4_NO_3_ proton-conducting SPE films with different concentrations of NH_4_NO_3_.

The values of S were derived from the slope of the linear part of logσ(ω) versus logω at higher frequencies region, as shown in Figure 6. In the present work, the acceptable frequency range is between 5.5<logω<6.5. The obtained values of S for pure PVA and PVA/NH_4_NO_3_ proton-conducting SPE films as a function of temperature are presented in Figure 7. For all presented samples, it is observed that the power-law exponent S decreases as temperature increases. It is also noted that the value of S is greater than unity at lower concentrations of NH_4_NO_3_ and low temperature, which suggests the occurrence of the ‘nearly constant loss’ phenomenon in these compositions. Generally, the nearly constant loss behavior in ionic conductors arises from the vibration of ions confined in the asymmetric double-well potential barrier, which correlated to the localized motions of charge carriers between neighboring sites rather than the hopping mechanisms in the conduction processes [46,47]. This phenomenon is observed for low-conducting SPEs at low temperatures, and cannot be observed when there are significant ion dynamics. Pal and Ghosh [48] have also reported the existence of a nearly constant loss at low temperatures in poly(methylmethacrylate)-lithium salt based polymer electrolytes plasticized with ethylene carbonate. They attribute the existence of this phenomenon to the cooperative hopping of charge carriers strongly coupled with the polymer chain dynamics.

Various theoretical models have been proposed to describe the conduction mechanism in electrolyte systems based on the variation of S with temperature. The most applicable models for SPEs are the correlated barrier hopping (CBH), the small polaron (SP), the overlapping large polaron (OLP), and the quantum mechanical tunneling (QMT) model [49]. According to the CBH model, S should decrease with increasing temperature, and the SP model is predominant if S increases with increasing the temperature. The OLP model is applicable if the S value decreases with increasing temperature to a minimum value and then increases again with further increases in temperature. Finally, the QMT model implies that S is temperature independent [7]. The continuous decrease in the S value with increasing temperature suggests that the CBH model is convenient to interpret the conduction mechanism in the present PVA/NH_4_NO_3_ proton-conducting SPE films, which means that the H^+^ ions can drift by hopping between adjacent hydroxyl groups of the PVA (complexation sites) over the potential barrier separating them [50].

#### 3.3.3. Bulk Conductivity

**Figure 8:**
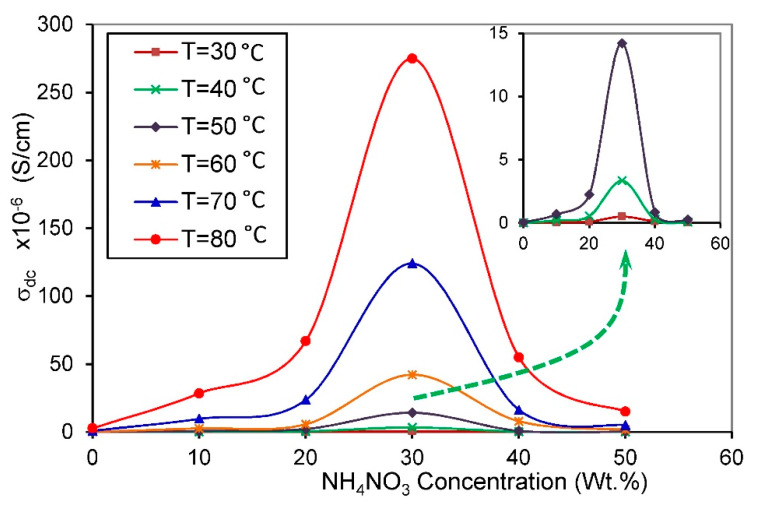
Conductance spectra for PVA/NH_4_NO_3_ proton-conducting SPE films with different salt concentrations at various temperatures.

It is well reported that bulk conductivity ($\sigma_{DC}$) can be calculated by extrapolating the frequency-independent plateau region of electrical conductivity towards zero frequency [32,51]. Figure 8 deals with the bulk conductivity of the PVA/NH_4_NO_3_ proton-conducting SPE system heated at different temperatures. When the temperature increases, the bulk conductivity improved for all concentrations, indicating that the conductivity is thermally assisted. The continuous increase in $\sigma_{DC}$ with increasing temperature for all compounds can be clarified based on the free-volume theory. As the temperature increase, the free-volume of the SPEs increase, causing the enhancement of the segmental motion of the chain, which facilitates the movement of ionic charge carriers [52].

The room temperature conductivity of pure PVA is 4 × 10^−9^ S/cm, which is compatible with the earlier reported value [53]. The electrical conductivity of SPE increased by several orders of magnitude with NH_4_NO_3_ loading. Among all SPE compositions, 30 wt.% of NH_4_NO_3_ doped polymer matrices show the maximum bulk conductivity in 5.17 × 10^−5^ S/cm at room temperature (303 K) and 2.74 × 10^−4^ S/cm at high temperature (353 K). This value is much higher than that achieved by Khandale et al. [54]; they observed maximum room temperature conductivity as 1.3 × 10^−7^ S/cm for PVA: ammonium acetate proton-conducting SPE.

The increment of NH_4_NO_3_ loading up to 30 wt.% caused an increase in the $\sigma_{DC}$, because of the rise in the number of free charge carriers (n) and their mobility (μ) since the DC conductivity is defined as the product of these two parameters, and the charge of the electron ($\sigma_{DC}=\sum en_{i}\mu_{i}$) [55]. The mobility of free ions in the SPE can be enhanced by increasing the amorphous fraction, which facilitates the movement of free ions by reducing the energy barrier prompting enhancement in the conductivity [32]. Recrystallization of the salt out of the film at high salt concentrations will contribute to reducing the number of mobile ions [56].

Above 30 wt.% of NH_4_NO_3_, the salt is no longer dissoluble within the matrix. This result is in concordance with XRD analysis, which demonstrates that no more of the salt can dissolve at a high concentration of NH_4_NO_3_.

From XRD results, the increase in electrical conductivity with increasing NH_4_NO_3_ content can be attributed to the reduction in the crystallinity phase of the host polymer matrix [57]. It is inferred that the SPE sample containing 30 wt.% of the salt is the most amorphous sample. The decreased trend in the conductivity at higher salt concentrations is because of the increase in the degree of crystallinity, as verified by the XRD result in Figure 2.

#### 3.3.4. Temperature-Dependent Conductivity

The temperature-dependent electrical conductivity may provide valuable information describing the ionic conduction behavior [55]. For all PVA/NH_4_NO_3_ proton-conducting SPE films, the conductivity increases exponentially with increasing the temperature due to the increase in the ion mobility. On the other hand, due to the complexity and the lack of correlation between the structure and transport properties of SPE systems, the ion transport in these systems cannot be comprehensively described [58]. Nevertheless, the temperature-dependent ionic conductivity of SPE was extensively studied using the experimentally Arrhenius and Vogel–Tammann–Fulcher (VTF) models [10].

The main factor influening the ionic conductivity (σ) of SPE is the activation energy ($E_{A}$), because it corresponds to the energy barrier for ionic conduction. It was observed that $E_{A}$ is inversely proportional to the conductivity of the system. The $E_{A}$ is influenced by three factors, i.e., the nature of bonds, vacancies, and site geometry [59]. The value of $E_{A}$ assesses the ionic mobility (μ) and charge carrier concentration (n). According to the Arrhenius equation, the activation energy ($E_{A}$) can be evaluated using the formula:

(4) $$\sigma =\sigma_{0}\exp\left(-\frac{E_{A}}{K_{\beta }T}\right)$$

where $\sigma_{0}$ is a pre-exponential factor, $K_{\beta }$ is the Boltzmann constant, and T is the absolute temperature. The Arrhenius equation describes ionic transport as intermolecular ion hopping in a dilute electrolyte [60]. In most cases, a SPE is more suitably fitted by the VTF equation, which can be expressed as the following equation [61]:

(5) $$\sigma =C/T^{1/2}\exp\left(-\frac{E_{P}}{K_{\beta }\left(T-T_{0}\right)}\right)$$

where C, $E_{P}$, $T_{0}$ are the constants determined through experimental data fitting. $E_{P}$ is the pseudo-activation energy, and $T_{0}$ is the reference temperature, which is usually equal or lower than the glass transition temperature by 10–50 K. The VTF behavior is correlated with the transport of mobile ions and the movement of long-range polymer chain segments. 

**Figure 9:**
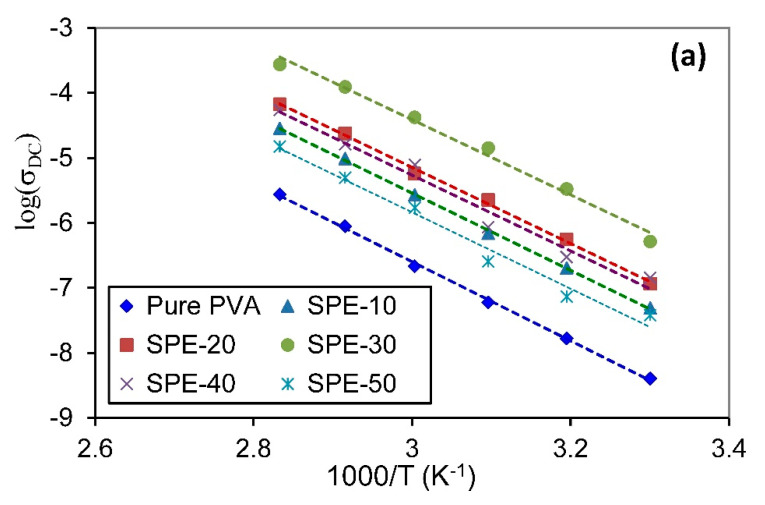

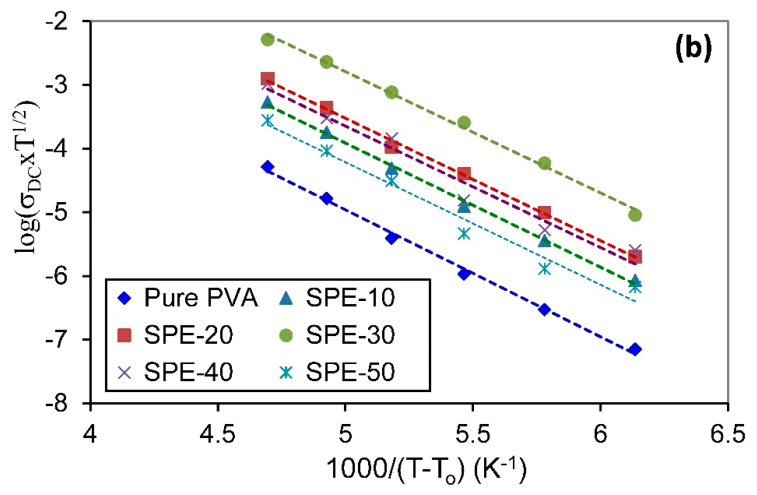
Temperature-dependence of DC conductivity for pure PVA and all PVA/NH_4_NO_3_ proton-conducting SPE films doped with various concentrations of NH_4_NO_3_, using (**a**) Arrhenius and (**b**) Vogel–Tammann–Fulcher (VTF) models.

In this study, both Arrhenius and VTF equations are used to model the conductivity of the present PVA/NH_4_NO_3_ proton-conducting SPE films for the sake of verification of the validity of two models. The parameters in Arrhenius and VTF equations could be attained by linearizing the fitting Equations (4) and (5), which are tabulated in Table 2. $T_{0}$ was obtained by trial and error to make the conductivity-temperature data fit Equation (2). In this study $T_{0}$ is taken to be 140 K. The distinction in the conductivity as a component of inverse temperature for pure PVA and all PVA/NH_4_NO_3_ proton-conducting SPE films are displayed in Figure 9.

The linear relationship of the $\sigma_{DC}$ versus the reciprocal temperature indicates that the $\sigma_{DC}$ increases exponentially with temperature, and the relaxation process is thermally activated [62]. The regression values ($R^{2}$) obtained for both models are close to unity, suggesting that all data points lie on the fitting straight lines. The values of activation energy ($E_{A}$) and pseudo-activation energy ($E_{P}$) were calculated from the slopes of the linear fits of Figure 9. The $E_{A}$ represents the minimum energy required for charge carriers to hop from one site to another site through the material, thereby causing electrical conduction [63]. While, $E_{P}$ is associated with the polymer segmental motion [55]. The values of $E_{A}$ and $E_{P}$ are least for the sample with the highest conductivity (SPE-30). Therefore, it can be concluded that the maximum conductivity of the SPE-30 sample was achieved by minimizing the energy barrier for ion migration.

## 4. Conclusions

A series of PVA/NH_4_NO_3_ proton-conducting SPE films were prepared by the solvent-casting process. The XRD patterns for SPEs confirm the reduction in the intensity of the peak corresponding to PVA upon an increase in the NH_4_NO_3_ content. The electrical conductivities were calculated, and it was found to increase from order 10^−9^ S/cm to 10^−5^ S/cm at room temperature, and 10^−4^ S/cm at 353 K. The highest ionic conductivity at ambient temperature was achieved for PVA loaded 30 wt.% NH_4_NO_3_ which are associated with the decrease in free ions and lowest degree of crystallinity. The temperature-dependent AC conductivity analysis infers the dominance of the correlated barrier hopping (CBH) model in the conduction mechanism of the system. The increase in conductivity with temperature was studied using both the Arrhenius and VTF models. In both cases, the maximum conducting sample exhibits the lowest activation energy and pseudo-activation energy, respectively. This observation indicates that the polymer segmental motions play a crucial role in the ion transport processes in the present SPE system.

## Figures and Tables

**Table 1:** Crystalline, total area, and degree of crystallinity ($\chi_{c}$ for pure PVA and PVA/NH_4_NO_3_ proton-conducting SPE films.

Samples	$A_{c}$	$A_{t}$	$\chi_{c}$
Pure PVA	9734.15	24,827.16	39.20
SPE-10	4864.19	27,295.78	17.82
SPE-20	7320.78	65,735.29	11.13
SPE-30	5410.93	54,434.50	9.94
SPE-40	7433.04	53,423.04	13.91
SPE-50	13,342.05	61,947.55	21.53

**Table 2:** Parameters determined through experimental data fitting using Arrhenius and Vogel–Tammann–Fulcher (VTF) models.

Samples	Arrhenius Model			VTF Model	
	$E_{A}\;\left(eV\right)$	$R^{2}$	$T_{0}\;\left(K\right)$	$E_{P}\;\left(eV\right)$	$R^{2}$
Pure PVA	1.207	0.9988	140	0.396	0.9959
SPE-10	1.179	0.9994	140	0.387	0.9973
SPE-20	1.161	0.9977	140	0.382	0.9974
SPE-30	1.147	0.9896	140	0.378	0.9954
SPE-40	1.156	0.9811	140	0.379	0.9684
SPE-50	1.166	0.9808	140	0.382	0.9745
